# The Healing Capability of Clove Flower Extract (CFE) in Streptozotocin-Induced (STZ-Induced) Diabetic Rat Wounds Infected with Multidrug Resistant Bacteria

**DOI:** 10.3390/molecules27072270

**Published:** 2022-03-31

**Authors:** Rewaa Ali, Tarek Khamis, Gamal Enan, Gamal El-Didamony, Basel Sitohy, Gamal Abdel-Fattah

**Affiliations:** 1 Department of Botany, Faculty of Science, Mansoura University, Mansoura 35516, Egypt; abdelfattaham@yahoo.com; 2 Department of Pharmacology, Faculty of Veterinary Medicine, Zagazig University, Zagazig 44519, Egypt; gamal.enan@zu.edu.eg; 3 Department of Botany and Microbiology, Faculty of Sciences, Zagazig University, Zagazig 44511, Egypt; eldidamonyg@gmail.com; 4 Department of Radiation Sciences, Oncology, Umeå University, SE-90185 Umea, Sweden; basel.sitohy@umu.se; 5 Department of Clinical Microbiology Infection and Immunology, SE-90185 Umea, Sweden

**Keywords:** diabetic foot ulcer, MDR-*Proteus mirabilis*, inflammatory markers, *Syzygium aromaticum*, growth factor

## Abstract

Treatment of diabetic foot ulcer (DFU) is of great challenge as it is shown to be infected by multidrug resistant bacteria (MDR bacteria). Sixty four bacterial isolates were isolated from DFU cases; antibiotic susceptibility tests were carried out for all of them. One bacterial isolate (number 11) was shown to resist the action of 8 out of 12 antibiotics used and was identified by both a Vitek-2 system and 16S rRNA fingerprints as belonging to *Proteus mirabilis,* and was designated *Proteus mirabilis* LC587231 (*P. mirabilis*). Clove flower extract (CFE) inhibited distinctively the *P. mirabilis* bacterium obtained. GC-MS spectroscopy showed that this CFE contained nine bioactive compounds. The effect of CFE on wound healing of Type 1 diabetic albino rats (*Rattus norvegicus*) was studied. The results indicated that topical application of CFE hydrogel improved wound size, wound index, mRNA expression of the wound healing markers (*Coli1, MMP9*, *Fibronectin*, *PCNA*, and *TGFβ*), growth factor signaling pathways (*PPAR-α*, *PGC1-α*, *GLP-1*, *GLPr-1*, *EGF-β*, *EGF-βr*, *VEGF-β*, and *FGF-β*), inflammatory cytokine expression (*IL8*, *TNFα*, *NFKβ*, *IL1β*, and *MCP1*), as well as anti-inflammatory cytokines (*IL4* & *IL10*), pro-apoptotic markers (*FAS*, *FAS-L*, *BAX*, *BAX/BCL-2*, *Caspase-3*, *P53*, *P38*), as well as an antiapoptotic one (*BCL2*). Furthermore, it improved the wound oxidative state and reduced the wound microbial load, as the cefepime therapy improved the wound healing parameters. Based on the previous notions, it could be concluded that CFE represents a valid antibiotics alternative for DFU therapy since it improves diabetic wound healing and exerts antibacterial activity either in vitro or in vivo.

## 1. Introduction

Diabetic foot ulcers and their infections are common complications associated with diabetic foot diseases; these complications are common cause of morbidity and impose a substantial burden on the patient and society [1]. The diabetic foot infection is eventually associated with an inflammatory response and tissue injury that could aggravate the clinical spectrum from simple, superficial cellulitis to chronic osteomyelitis [2]. A significant problem facing physicians in the treatment of diabetic foot ulcer (DFU) is the existence of multidrug-resistant (MDR) microbes, where many variants of MDR microbes have been isolated and characterized [3,4,5,6,7,8,9,10,11]. MDR-*P. mirabilis* is one of the most prevalent microbes to exist in diabetic foot ulcers (DFU) [12]. There is a vast need to search for other innovative therapies based on using natural plant extract either singly or in combination with antibiotics [11], probiotics [3,13,14,15,16,17], natural and modified proteins [18,19], nanoparticles [20], and phage therapy [21].

Peripheral diabetic complications, such as neuropathy, angiopathy, and the infections of the DFU extend the inflammatory stage with the overexpression of inflammatory cytokines such as *IL-1β* [22], *IL-6* [23], *TNF-α* [23], *NF-κβ* [24], and *MCP-1* [25], which increase the tissue damage, imposing either a proliferative or maturation stage, which results in retarding the diabetic wound healing [26]. Several medicinal herbal extracts have achieved reasonable therapeutic goals regarding DFU infection [27]. *S. aromaticum* is one of those herbs, belong to the family Myrtaceae, and exerts broad-spectrum antibacterial activity against MDR bacteria [28] and has other beneficial biological activities such as anti-inflammatory, antioxidant, and antibacterial effects [29]. The anti-inflammatory effect was recognized as one of the most beneficial effects regarding the medicinal plant that reduces the unnecessary inflammatory response, thus, preventing further tissue damage and increased expression of T helper lymphocytes (Th2), which regulate the inflammatory process and minimize the unnecessary inflammatory cascade [30]. *S. aromaticum* is traditionally used in the treatment of wounds, burns, and tooth affection as a pain reliever, as well as treating tooth infection and toothache. Additionally, it is used in several industrial processes such as soap, perfumes, and in cleaning vehicles [31]. Interestingly, CFE used in Indian and Chinese traditional medicine [32]. CFE contains several bioactive compounds which are known for their anti-inflammatory effects, via switching of TNF-α inflammatory cascades and inducing Nrf-2, which is considered the major anti-inflammatory pathway [33]. Also, they exert a broad antibacterial spectrum against several pathogens [34]. Several studies published recently used topical hydrogel-based plant extracts in treatment of either skin infections or as having wound healing potential. For instance, Antonescue et al. [35,36] studied the pharmacological effects of *Ocimum basilicum* and *Trifolium pratense* either singly or in combination of both of them; they showed to be superior in their wound healing potential.

The present study was designed to investigate the phytochemical profile of the CFE and its therapeutic potential in the treatment of a multidrug-resistant *P. mirabilis* LC587231-infected diabetic excisional wound model as an antibiotic alternative for DFU therapy, as well as to study the underlying mechanisms for the healing process.

## 2. Results

### 2.1. Characterization of the Bacterial Isolates Obtained and Their Antibiotic Bioassay

Gram staining was carried out for the 64 bacterial isolates obtained. About 33 bacterial isolates and 31 bacterial isolates were G^+^ and G^−^ bacteria, respectively (Supplementary Table S1). The antibiotic sensitivity test was carried out for those 64 bacterial isolates. Results are given in Supplementary Table S1. Except for the bacterial isolates DFU 9, 11, 16, and 32, all isolates showed variability in their sensitivity or resistance to the 13 antibiotics investigated; such bacterial isolates DFU 9, 11, 16, and 32 were showed to be multidrug resistant as they possessed higher MAR indices of about 30.7, 53.8, 23.1, and 23.1%, respectively (Supplmentary Table S1). The MDR isolates DFU 9, 11, 16, and 32 were identified by a Vietek-2 system and were shown to be strains belonging to *Morganella morgani*, *Proteus mirabilis*, *Serrataia fonticola*, and *Escherichia coli,* respectively (Table 1). *P. mirabilis* DFU11 was of interest as it was dominant within the bacterial isolates obtained and possessed the highest MAR index. Consequently, it was identified at the molecular level by the sequencing of 16S rRNA which confirmed the biochemical identification carried out by the Vitek-2 system. The bacterium *P. mirabilis* was deposited in the NCBI with accession number LC587231 (Supplementary Figure S1). Since *P. mirabilis* LC587231 resisted the action of ampicillin, ampicillin/sulbacreram, aztreonam, cefazolin, cefepime, ceftriaxone, tigecycline, and nitrofuran, it was used for further work in this study.

The antibiotic cefepime is one of choice in treatment of diabetic food infections in Egypt and it is available in Egyptian market. Consequently, the inhibition of *P. mirabilis* isolated from diabetic foot biopsy by higher concentrations of cefepime was a necessary goal. Thus, the MIC of the antibiotic cefepime was determined against the *P. mirabilis* bacterium isolated from diabetic foot biopsy. The *P. mirabilis* was resistant to 10–49 μg/mL cefepime. MIC was shown to be 50 μg/mL and MBC was 60 μg/mL (Table 2). Therefore, higher concentrations of the antibiotic cefepime were used in further experiments. About 20 mg/mL (1 g/50 mL) was used, as it is available and prepared in Egyptian pharmacies.

### 2.2. Phytochemical Screening of CFE and In Vitro Antibacterial Activity

**Figure 1:**
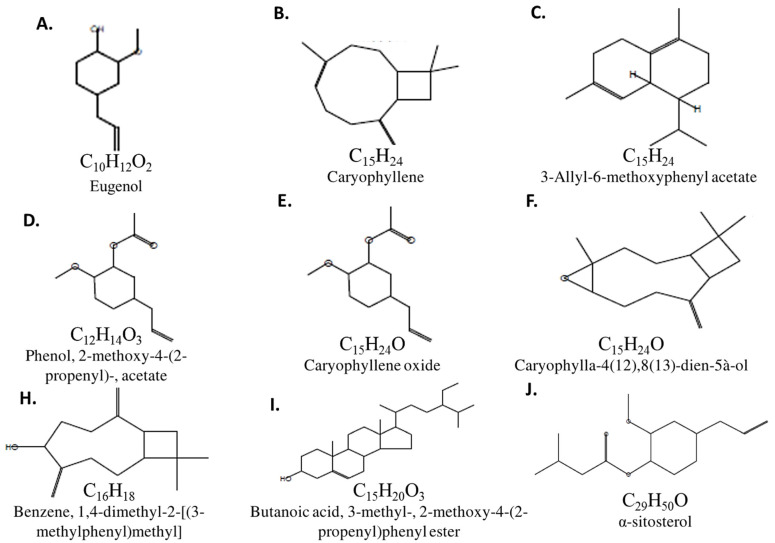
Chemical structure of the GC-MASS-screened *Syzygium aromaticum* ethanolic extract bioactive compounds (**A**–**J**).

The GC-Mass running times for CFE were 57 min. The GC-Mass phytochemical screening of CFE was done with the aid of the National Institute Standard and Technology (NIST). The spectrum of the screened unknown compounds were compared with the NIST stored known spectra and revealed nine bioactive compounds (Table 3 and Figure 1). Supplementary data (Figures S2 and S3) show that eugenol represented the higher abundance among the identified bioactive compounds (Table 3 and Supplementary data (Figures S2 and S3)). Moreover, CFE showed in vitro antibacterial activity against *P. mirabilis*, as inhibition zones of about ≥20 mm were detected in lawns of *P. mirabilis* around wells containing CFE. As given in Table 2, the MIC CFE was 8 μg/mL. Minimum bactericidal concentration (MBC) was 10 μg/mL.

### 2.3. Validation of Type 1 Diabetic and Diabetic Wound Onset

**Figure 2:**
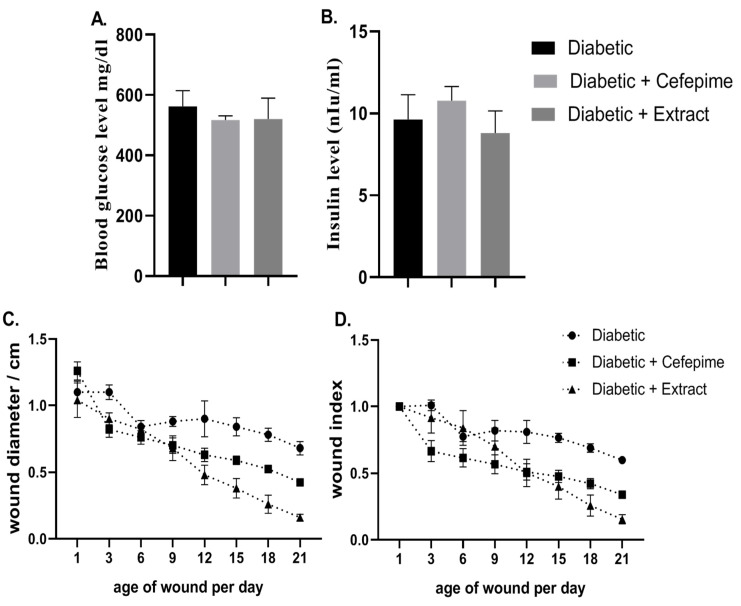
Glycemic (**A**,**B**) and wound parameters of Type 1 diabetic rats throughout 21 days. Fasting blood glucose level (mg/dL) (**A**), serum insulin level (mIU/I) (**B**), wound diameter (cm) (**C**) and wound index (**D**).

**Figure 3:**
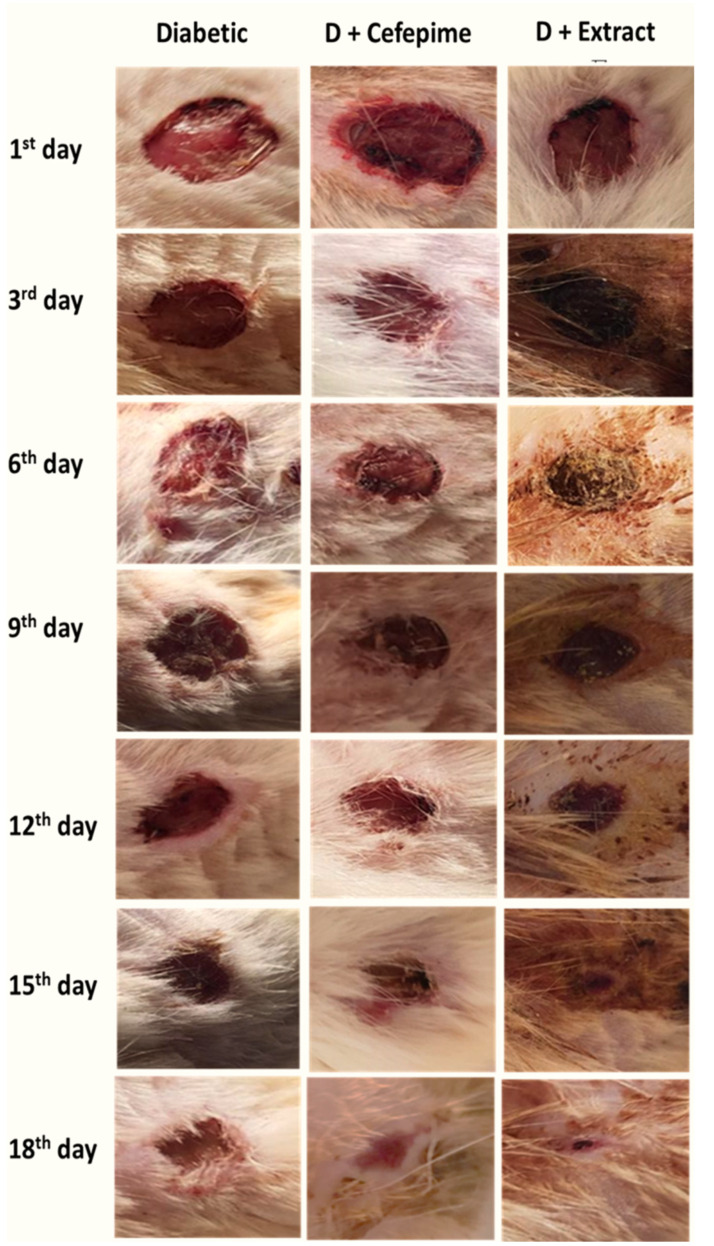
Wound imaging follow-up on the 1st, 3rd, 6th, 9th, 12th, 15th, and 18th days. (three days post diabetic wound curation and infection with *P. mirabilis*).

The results of the present investigation illustrated that the streptozotocin (STZ), intraperitoneally injected in rats, lead to the development of both sustained elevations in blood glucose level ≥500 mg/dL and a sharp decrease in serum insulin level <3.5 mIU/mL (Figure 2A,B) with a progressive decrease in body weight within the first 3 weeks of diabetic induction. This was correlated with a clear manifestation of polydipsia, polyuria, monitored with water intake and little moisture content, and polyphagia, monitored with daily feed intake of the rats investigated. Moreover, three days post diabetic wound curation and infection with *P. mirabilis*, the wound showed signs of suppuration and local bacterial infection without the development of any signs of systemic bacterial infection (Figure 3).

### 2.4. Effect of CFE and Cefepime on Wound Healing

It was illustrated that both CFE and cefepime topical application caused a significant (*p* < 0.001) reductions in the mean value of wound size and wound index when compared with diabetic rats with the rank of CFE, followed by the cefepime-treated group (Figure 2C,D and Figure 3). 

### 2.5. Effect of CFE and Cefepime on the Expression of Wound Healing Markers and Collagen Deposition

**Figure 4:**
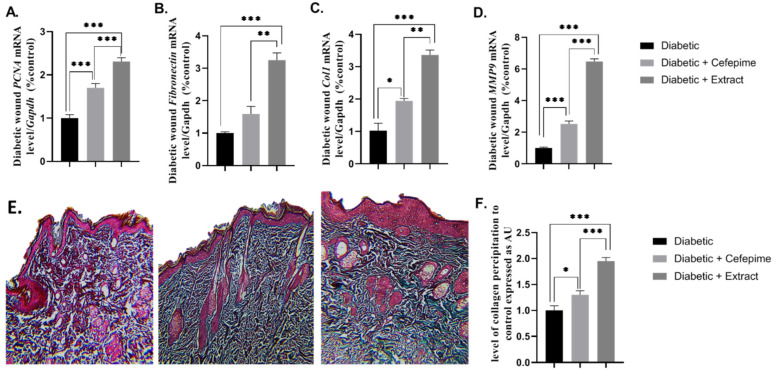
Effect of the hydrogel topical application of cefepime and CFE once daily for 14 successive days on the mean fold change of the, mRNA relative expression of regenerative markers (*PCNA*, *Fibronectin*, *COL1*, *and MMP9*) to internal control gene *Gapdh* (**A**–**D**) and collagen deposition (**E**,**F**) of a diabetic wound infected with clinical isolates of *P. Mirabilis* infected with LC587231 in Type 1 diabetic rats. (**A**) *mRNA* relative expression of *PCNA* to internal control gene *Gapdh*, (**B**) mRNA relative expression of *Fibronectin* to internal control gene *Gapdh*, (**C**) *mRNA* relative expression of *COL1* to internal control gene *Gapdh*, (**D**) *mRNA* relative expression of *MMP9* to internal control gene *Gapdh*, (**E**) a photomicrograph of Masson blue staining of: control group (left panel), cefepime-treated group (middle panel), and CFE-treated group (right panel), and (**F**) for the level of the collagen deposition.

The results obtained herein indicated that the topical application of CFE significantly upregulated the mean fold change of the mRNA expression of the healing biomarkers: *PCNA*, *Collagen-1*, *MMP-9*, *Fibronectin*, and collagen either compared with the diabetic group (*p* < 0.001) or cefepime-treated group (*p* < 0.01). However, the cefepime-treated group showed a significant upregulation in the mean fold change of the mRNA expression of *fibronectin*, *MMP-9* (*p* < 0.001), and *coli1* (*p* < 0.05) when compared with the diabetic group (Figure 4A–F).

### 2.6. Effect of Topical Application of Either CFE or Cefepime Hydrogel on the Wound Growth Factors Signaling Pathway

**Figure 5:**
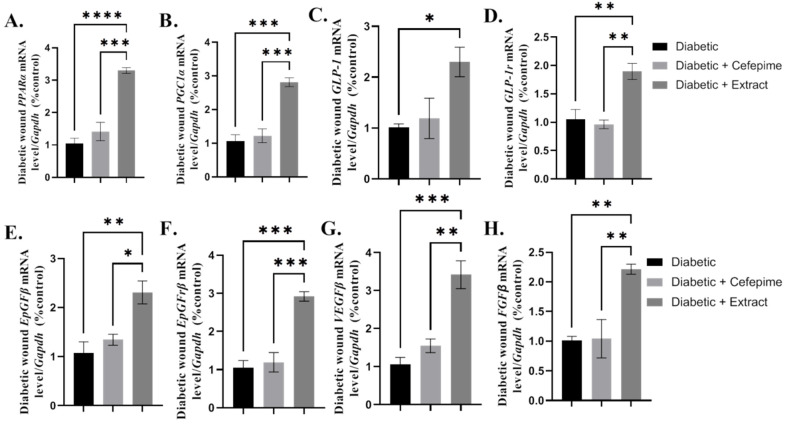
Effect of the hydrogel topical application of cefepime and CFE once daily for 14 successive days on the mean fold change of the *mRNA* relative expression of growth factors signaling pathway (*PPAR-α*, *PGC1-α*, *GLP-1*, *GLPr-1*, *EGF*, *EGFr*, *VEGF*, and *FGF*) to internal control gene *Gapdh* (**A**–**D**) and collagen deposition (**E**,**F**) of a diabetic wound infected with *P. Mirabilis* in Type 1 diabetic rats. (**A**) *mRNA* relative expression of *PPAR-α* to internal control gene *Gapdh*, (**B**) *mRNA* relative expression of *PGC1-α* to internal control gene *Gapdh*, (**C**) *mRNA* relative expression of *GLP-1* to internal control gene *Gapdh*, (**D**) *mRNA* relative expression of *GLPr-1* to internal control gene *Gapdh*, (**E**) *mRNA* relative expression of *EGF* to internal control gene *Gapdh*, (**F**) *mRNA* relative expression of *EFGr* to internal control gene *Gapdh*, (**G**) *mRNA* relative expression of *VEFG* to internal control gene *Gapdh,* and (**H**) *mRNA* relative expression of *FGF* to internal control gene *Gapdh*. **** very distinctive difference; *** Distinctive difference; ** Moderate difference; * Scant difference.

The results of the present investigation showed that CFE (topical application) significantly upregulated the *mRNA* expression of *PPAR-α* (*p* < 0.0001), *PGC1-α* (*p* < 0.0001), *GLP-1* (*p* < 0.05), *GLPr-1* (*p* < 0.05), *EPGF-β* (*p* < 0.001), *EPGFr* (*p* < 0.0001), *VEGF-β* (*p* < 0.0001), and *FGF-β* (*p* < 0.05) both when compared with the diabetic group or cefepime-treated one. Additionally, cefepime topical application showed upregulation of those mRNA expression factors that matched that obtained by CFE; both CFE and cefepime showed better effects when compared with controls (Figure 5A–H).

### 2.7. Effect of CFE and Cefepime on the Wound Oxidative Status

**Figure 6:**
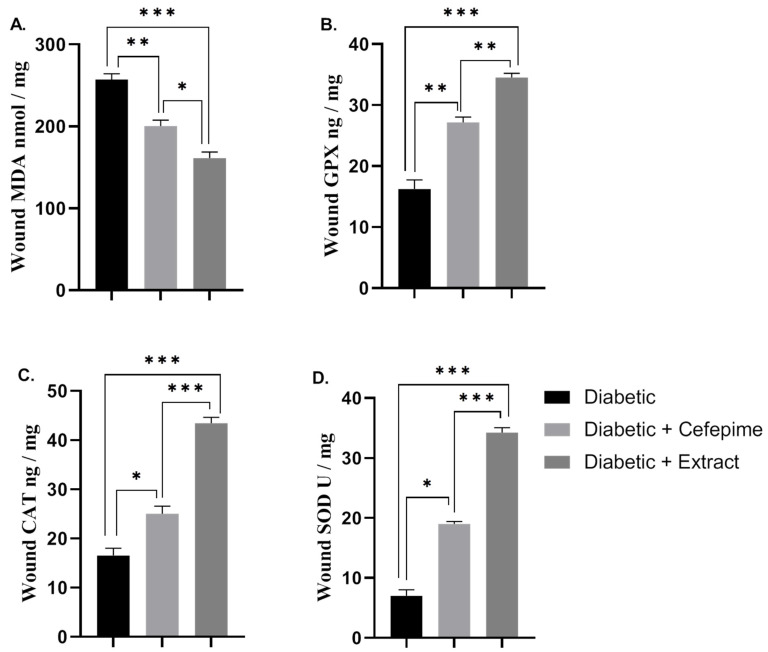
Effect of the hydrogel topical application of cefepime and CFE once daily for 14 successive days on the mean value of the oxidative status of the diabetic wound infected with *P. Mirabilis* in Type 1 diabetic rats (**A**–**D**). (**A**) lipid peroxidation marker (MDA nmol/mg), (**B**) GPx ng/mg, (**C**) CAT ng/mg, (**D**) SOD U/mg. *** Distinctive difference; ** Moderate difference; * Scant difference.

The results of this investigation (Figure 6A–D) have showed that topical application of CFE caused a significant decrease (*p* < 0.001) in the mean value of the lipid peroxidation marker malondialdehyde (MDA) and a significant increase (*p* < 0.001) in the mean value of the antioxidant enzyme activity of glutathione peroxidase (GPx), superoxide dismutase (SOD), and common antioxidant enzyme (CAT) either compared with the diabetic group (control) or the cefepime-treated one. Moreover, antibiotic therapy showed a significant increase in the mean value of the diabetic wound antioxidant activity either for GPx (*p* < 0.01), or CAT, or SOD (*p* < 0.05). Additionally, there was a significant decrease in the mean value of lipid peroxidation markers MDA (*p* < 0.01) when compared with the diabetic group (Figure 6A–D).

### 2.8. Effect of CFE and Cefipime on the Expression of Wound Inflammatory and Anti-Inflammatory Markers

**Figure 7:**
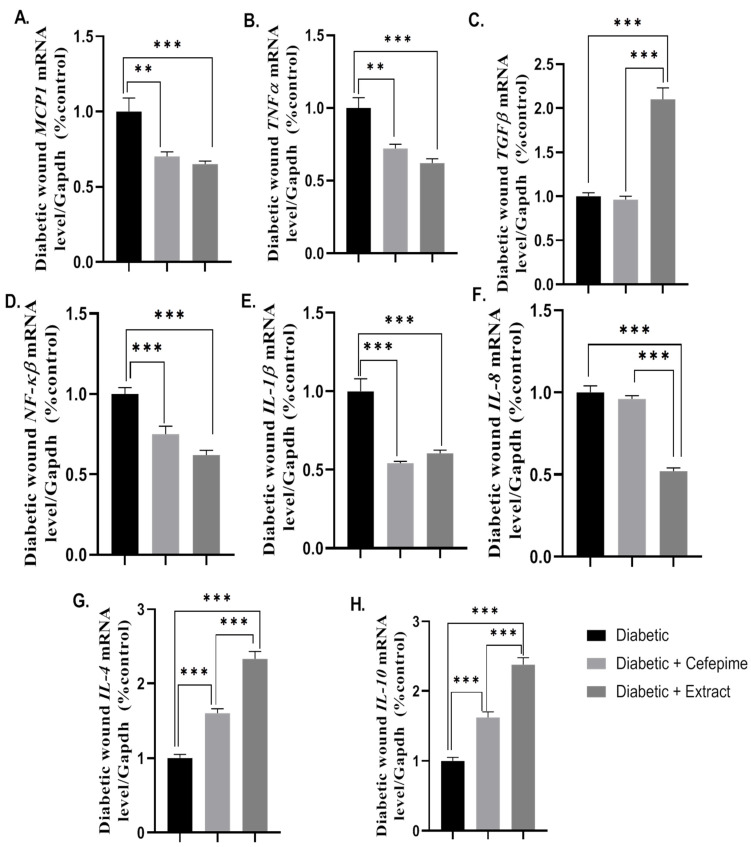
Effect of the hydrogel topical application of cefepime and CFE once daily for 14 successive days on the mean fold change of the *mRNA* relative expression of inflammatory and anti-inflammatory markers to internal control gene *Gapdh* of the diabetic wound infected with *P. Mirabilis* in type 1 diabetic rats (**A**–**H**). (**A**) *mRNA* relative expression of *MCP1* to internal control gene *Gapdh*, (**B**) *mRNA* relative expression of *TNF-α* to internal control gene *Gapdh*, (**C**) *mRNA* relative expression of *TGF-β* to internal control gene Gapdh, (**D**) *mRNA* relative expression of *NF-κβ* to internal control gene *Gapdh*, (**E**) *mRNA* relative expression of *IL-1β* to internal control gene *Gapdh*, (**F**) *mRNA* relative expression of *IL-8* to internal control gene Gapdh, (**G**) *mRNA* relative expression of *IL-4* to internal control gene *Gapdh*, and (**H**) *mRNA* relative expression of *IL-10* to internal control gene *Gapdh*. *** Distinctive difference; ** Moderate difference.

Topical application of CFE on induced diabetic wounds showed a significant downregulation in the mean fold change of the mRNA expression of the inflammatory biomarkers (*p* < 0.001) *MCP1*, *TNF-α*, *NFKβ*, *IL-1β*, and *IL-8,* as well as a significant (*p* < 0.001) upregulation in the mean fold change of the mRNA expression of anti-inflammatory markers such as *IL-10* and *IL-4,* and a pro-inflammatory one, *TGF-β,* when compared to the diabetic group as well as for the cefepime-treated group for all the aforementioned markers, with the exceptions of *IL-8* and *TGF-β,* which appeared not affected. Interestingly, CFE topical application showed significant (*p* < 0.001) improvements in the mRNA expression of the anti-inflammatory markers *IL-10* and *IL-4* when compared to the cefepime-treated group (Figure 7A–H). 

### 2.9. Effect of CFE and Cefepime on the Wound Apoptotic Signaling Pathway

**Figure 8:**
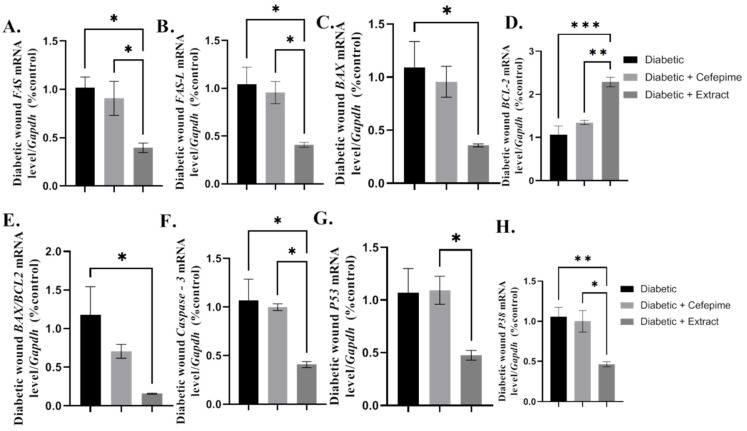
Effect of the hydrogel topical application of cefepime and CEF once daily for 14 successive days on the mean fold change of the *mRNA* relative expression of the apoptosis signaling pathway (*FAS*, *FAS-L*, *BAX*, *BCL-2*, *BAX/BCL-2*, *Caspase-3*, *P53*, and *P38*) to internal control gene *Gapdh* (**A**–**D**) and collagen deposition (**E**,**F**) of diabetic wound infected with clinical isolates of *P. Mirabilis* infected LC587231 in type 1 diabetic rats. (**A**) *mRNA* relative expression of *FAS* to internal control gene *Gapdh*, (**B**) *mRNA* relative expression of *FAS-L* to internal control gene *Gapdh*, (**C**) *mRNA* relative expression of *BAX* to internal control gene *Gapdh*, (**D**) *mRNA* relative expression of *BCL-2* to internal control gene *Gapdh*, (**E**) *mRNA* relative expression of *BAX/BCL-2* to internal control gene *Gapdh*, (**F**) *mRNA* relative expression of *Caspase-3* to internal control gene *Gapdh*, (**G**) *mRNA* relative expression of *P53* to internal control gene *Gapdh,* and (**H**) *mRNA* relative expression of *P38* to internal control gene *Gapdh*. *** Distinctive difference; ** Moderate difference; * Scant difference.

The results of the current work illustrated that CFE topically treated diabetic wounds elicited a significant downregulation in the mean value relative mRNA expression of the pro-apoptotic markers *FAS* (*p* < 0.05), *FAS-L* (*p* < 0.05), *BAX* (*p* < 0.05*)*, *BAX/BCL-2* (*p* < 0.05), *Caspase-3* (*p* < 0.05), *P53* (*p* < 0.05), and *P38* (*p* < 0.05), as well as a significant (*p* < 0.001) upregulation in the mean fold change of the relative mRNA expression of the antiapoptotic one *BCL-2* (Figure 8A–H).

### 2.10. Effect of CFE and Cefepime on the Histopathological Picture of Type 1 Diabetic Wound

**Figure 9:**
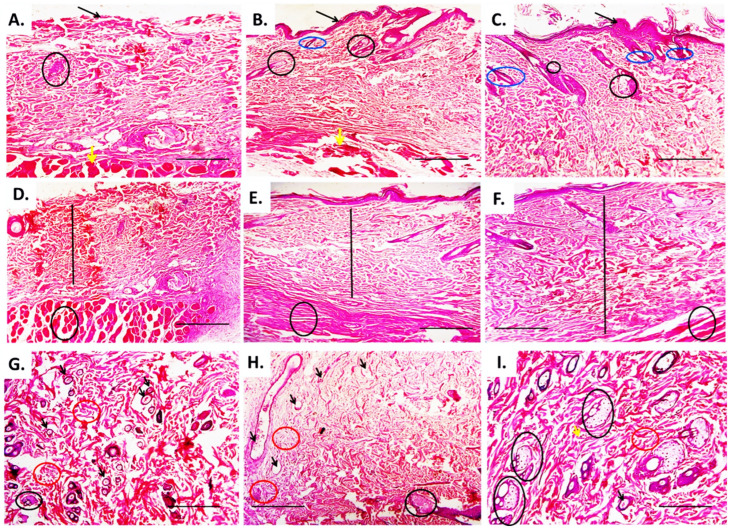
Effect of the hydrogel topical application of cefepime and CFE for successful 14 days on the histopathological changes of the clinical isolates of *P. mirabilis*-infected diabetic wound in rats (**A**–**I**). (**A**) a photomicrograph of the diabetic infected untreated wound showing sloughed epidermal layer (black arrow), degenerated sebaceous gland (black circle), and smooth muscle (yellow arrow) H&E ×100, (**B**) a photomicrograph of cefepime-treated diabetic infected wound showing indefinite thin epidermal layer (black arrow), few degenerated sebaceous glands (black circle) and hair follicles (blue circle), and smooth muscle (yellow arrow) H&E, ×100, (**C**) a photomicrograph of clove ethanolic-extract treated diabetic infected wound showing thick well defined multicellular epidermal layer (black arrow), numerous regenerated hair follicles (blue arrow) and sebaceous glands (black circle), and well defined smooth muscle fiber (yellow arrow), H&E, ×100, (**D**) a photomicrograph of diabetic infected untreated wound showing narrow dermal layer with few depositions of collagen and granulation tissue (black line) and a severely degenerated muscular layer (black circle), H&E, ×100, (**E**) a photomicrograph of diabetic cefepime-treated diabetic infected wound showing moderate; dermal thickness (black line) and muscular degeneration (black circle), H&E, ×100, (**F**) a photomicrograph of clove ethanolic extract-treated diabetic wound showing heavy deposition of both collagen and granulation tissue with increasing dermal thickness (black line) and unaffected muscular tissue (black circle), H&E, ×100, (**G**) a photomicrograph of untreated diabetic infected wound showing severely congested blood vessels (black arrow), degenerated sebaceous glands (black circle), and abundant leukocytic infiltration with few fibroblastic ones (red circle), H&E, ×100, (**H**) a photomicrograph of cefepime-treated diabetic wound showing moderate blood vessel congestion (black arrow), leukocytic infiltration (red circle), and few sebaceous glands, H&E, ×100, (**I**) a photomicrograph of clove ethanolic extract-treated diabetic infected wound showing mild leukocytic infiltration (red circle), blood vessel congestion (black arrow), and numerous sebaceous glands with obvious filtration of fibroblast cells (yellow arrow), H&E, ×100.

Histopathological examination of the hematoxylin and eosin (H&E) stained section of the diabetic, infected, non-treated wound showed congested blood vessels, massive leukocytic infiltration, and appearance of vascular granulation tissue (Figure 9A,D,G). However, the histomorphic picture of the cefepime-treated diabetic wound indicated moderate leukocytic infiltration with the appearance of the epidermal pads, hair follicle, and collagen that indicated the common signs of wound healing (Figure 9B,E,H). On the other hand, histomorphological examination of CFE-treated diabetic wounds illustrated a mild inflammatory reaction, appearance of a moderate number of the fibroblast, along with granulation tissue, development of epidermal pads, increasing both epidermal and dermal thickness, and restoration of both the hair follicles and the sebaceous gland that indicated an advanced regenerative state of the damaged diabetic skin (Figure 9C,F,I).

## 3. Discussion

DFUs are most serious diabetic complications in Egypt; their infectious bacteria were found to develop resistant variants which make their treatment difficult [37]. This has resulted in interest in isolation and characterization of MDR bacteria infecting diabetic feet. In this study, about 64 bacterial isolates were isolated from DF biopsy; the MDR bacterial isolates 9, 11, 16, and 32 were identified by a Vitek-2 system as belonging to *Morganella morgani*, *Proteus mirabilis*, *Serratia fonticola*, and *Escherichia coli,* respectively. Many previous studies have identified clinical bacteria by the Vitek-2 system [21]. *P. mirabilis* showed the highest MAR index and was resistant to eight antibiotics out of the thirteen used. It was resistant to ampicillin, ampicillin/sulbactam, aztreonam, cefazoline, cefepime, ceftriaxone, tetracycline, and nitrofuran. This showed that the bacterium *P. mirabilis* was of interest and indicated a full characterization of this pathogen, its identification by 16S rRNA was done and confirmed a successful biochemical identification [16]. It was stored in Gene Bank under accession number LC5877231 and was designated *P. mirabilis* LC587231 (*P. mirabilis*).

The antibiotic cefepime is the one of choice in Egypt as it is available in the Egyptian market. *P. mirabilis* was resistant to cefepime discs containing 30 μg/mL and many infectious bacteria resist these antibiotics at lower concentrations [38]. The DF pathogen *P. mirabilis* was grown in different concentrations of the antibiotic cefepime and the MIC and MBC were shown to be 50 μg/mL; and 60 μg/mL, respectively. The *P. mirabilis* pathogen was resistant to 30 μg/cefepime disc and started to be inhibited at ≥50 μg/mL. This would be due to lower concentrations of the antibiotic cefepime not saturating the specific site(s) receptors of bacterial cell membrane and the higher concentrations might saturate these receptors and in turn interfere with bacterial cell wall synthesis by covalently binding enzymes, leading to bacterial inhibition [39,40].

CFE inhibited *P. mirabilis* distinctively in vitro and this concurs with latter published results [31]. In view of bioactive compounds elucidated by GC-Mass spectroscopy, almost all of them were reported to inhibit bacterial pathogens by different mechanisms of action. Eugenol, esters (4-alkyl-3-methoxy-phenyl acetate, 3-methyl-2-methoxy-4-(2-propenyl) phenyl butaneate), and α-sitosterol are, in general, positively charged and more hydrophobic and this leads to electrostatic interactions with bacterial cellular components, which could lose electrolytes, resulting in cell death [41]. Other heterocyclic compounds such as caryophyllenes and hexahydronaphthnlene appeared to herein inhibit pathogenic bacteria as they can interact either as electrophiles or nucleophiles of the cells, leading to inhibition of DNA synthesis, which causes cell death [11]. Finally, the bicyclic alcohol makes an unsuitable environment in which bacteria cannot grow [11].

Several types of proteins were responsible for wound healing such as *MMP3*, *MMP9*, collagen, and fibronectin that are secreted from both keratinocytes and fibroblasts [42], The present investigation revealed that the topical application of CFE elicited a significant improvement in wound size and wound index in comparison to both the antibiotic-treated group and control one [43]. On the other hand, the CFE-treated group showed a significant upregulation in the mRNA expression of the wound healing markers *Coli-1*, *MMP-9*, and *fibronectin* that go hand in hand with Galehdari et al. [44]. The above-mentioned data indicated an active wound repairing mechanism, since the *MMP9* activated the keratinocytes’ migration and secretion of *MMP3* is considered the most essential element for wound closure [45]. Moreover, one of the most recorded diabetic drawbacks is peripheral neuropathy and angiopathy that retard diabetic wound healing via interfering with wound peripheral circulation, thus, depriving the fibroblasts and the keratinocytes of the essential nutrient for their growth and proliferation [46]. The results of the present study showed that the topical application of CFE hydrogel induced a marked upregulation in the relative expression of the growth factors related to both angiogenesis, keratinocytes, fibroblast growth, and proliferation, which could be attributed to the increased expression of the glucagon-like peptides and their receptors, thus potentiating secretion of the growth factors such as vascular endothelial growth factors (VEGF) that improve wound microcirculation [47], epidermal growth factor (EPGF) [48], and fibroblast growth factors [49]. On the other hand, several previous reports illustrated the role of peroxisome proliferator-activated receptors alpha (PPAR-α) and their co-activators (PGC1-α) in the wound healing process [50,51,52].

The findings of the current work are in accordance with previous notions, as CFE application upregulated the expression of the *PPAR-α* and its coactivator *PGC1-α*. Besides, the hyperglycemic state that is experienced in Type 1 diabetes leads to an increase in advanced glycation products (AGP), reactive oxygen species (ROS), and reactive nitrogen species (RNS) that elicit an imbalance in the oxidant/antioxidant activity [53]. The findings of the current investigation were in the same line with the aforementioned consequences, illustrated by the marked increase in the lipid peroxidation marker MDA and decrease in the antioxidant activity for GPx, SOD, and CAT activity. Furthermore, the increased AGP, ROS, and NRS activates TNFα, inducing cleavage for the TNF superfamily secreted from the inflammatory cells [54,55], and stimulating NF-κβ, which impairs both angiogenesis and wound closure [56].

Interestingly, the results of the present investigation were in line with the previous notion since the infected diabetic wound showed a marked upregulation in the inflammatory marker mRNA expression of *IL-8*, *IL-1β*, and *TNF-α* as a response to the wound oxidative state that discussed the retarding of diabetic wound healing. However, topical application of the CFE on the diabetic infected wound improved the cellular events that were shown with the enhanced wound oxidative microenvironment, noticeably through downregulation of the inflammatory markers *TNF-α*, *IL-1β*, and *IL-8,* with a marked upregulation in the anti-inflammatory markers *IL-4* and *IL-10* [57]. The anti-inflammatory microenvironment created with topical application of CFE could be referred to by the bioactive compounds that are present in CFE [58,59], which inhibit MAPkinases (p38 pathway) and stimulate *IL-4* and *IL-10* anti-inflammatory cytokines [60]; likewise, evoked marked upregulation in the Nrf-2 that inhibits keap1 protein expression leads to induction of the AMPK/GSK3β/ERK pathway that downregulates expression of the NF-κβ-65, improving the wound microenvironment and reducing further tissue damage. The previous reports were in fulfillment of the results of the current study, as the diabetic infected wound showed upregulation in the mRNA expression of *MCP-1* that was downregulated in the CFE-treated group, followed by the antibiotic-treated one, which could explain the superior healing potency in the CFE group when compared with the others [61]. On the same line, *TGF-β* is considered one of the essential wound healing elements that is implicated in migration and differentiation of the fibroblast and endothelial cells, and improves wound healing, collagen, matrix metalloproteinase deposition, and angiogenesis [61].

The results employed herein showed that CFE-treated group showed a marked upregulation in the wound expression of *TGF-β1*; the retardation in the diabetic wound could be owed to the hyperglycemic wound oxidative stress that manifested with increasing AGE, ROS, and RNS, which stimulated mitogen-activated protein kinase/inflammatory/apoptotic pathways [62]. The CFE hydrogel topical application improved the wound healing, resulting in downregulation of the MAPK, apoptotic markers’ expression, and upregulation of the antiapoptotic marker *BCL- mRNA* expression, which was in line with Medicherla et al. [63], who reported that inhibition of the MAPK (P38) improves diabetic wound healing and re-epithelialization via modulation of inflammatory and apoptotic signaling pathways. These data evidenced that CFE comprised bioactive compounds that abolished the diabetic cytotoxic effect on the keratinocytes and fibroblast.

In regard to the total bacterial count and the coliform count, they were significantly reduced in both the CFE-treated group and the cefepime-treated one when compared with the diabetic infected group (control), which might reveal that CFE contains antibacterial bioactive compounds [64].

Finally, the results of the present work showed that the use of CFE almost matched the use of the cefepime antibiotic concerning infected diabetic wound healing, which might be ascribed to the cytotoxicity of the antibiotics to both fibroblasts and keratinocytes, reducing the proliferative capacity, collagen, fibronectin, and wound matrix protein deposition, forming either less tensile granulation tissue or scar formation [65,66,67,68]. However, the antibiotics could improve diabetic wound healing via controlling the bacterial infection, increasing the innate immune cells infiltration, but unfortunately, they negatively impacted the fibroblast and keratinocytes’ viability, proliferative capacity, and, consequently, the matrix proteins’ formation and re-epithelialization that was shown with the reduced expression of the *PCNA* and the upstream expression of the apoptotic pathway. Such limitations could be avoided with *Syzygium aromaticum* extract application that comprises both antibacterial activity and wound remodeling capabilities, without neither fibroblast nor keratinocytes cytotoxicity.

Further work will be necessary to investigate the effect of combinations of the antibiotic cefepime and CFE on the inhibition of this *P. mirabilis* and other MDR bacteria in vitro and in vivo.

## 4. Materials and Methods

### 4.1. Chemicals, Drugs, and Plants

Chemicals for diabetes induction (STZ, citric acid, sodium citrate), and hydrogel formation (sodium metabisulfite, sodium methylparaben, sodium propylparaben, and carbopol 934) were purchased from Sigma Aldrich (USA). Cefepime antibiotics were provided from Pharco Pharmaica Company, Cairo, Egypt. The plant clove (*S. aromaticum*) was obtained from a herbal market at Mansoura city (100 km North Cairo, Egypt), identified morphologically in the Department of Botany Faculty of Science Mansoura University, Egypt, and the plant name was checked with http://www.theplantlist.org (accessed on 2 March 2022) and http://ipni.org/urn:lsid:ipni.org:names:601421-1 (accessed on 2 March 2022). Additionally, the clove is listed in the European pharmacopeia (EMA/HMPC/475451/2020 & EMA/HMPC/534924/2010 & EMA/HMPC/534946/2010 & EMA/HMPC/534948/2010).

### 4.2. Pus Sample Collection and Bacterial Identification

A total of forty-seven diabetic patients’ pus samples were collected from Zagazig University hospital (Zagazig, 80 km North Cairo, Egypt), with the patient’s written informed consent. Pus samples were transported to the Laboratory of Microbiology, Faculty of Science, Zagazig University in a sterile containers within one hour for microbiological analysis. The bacterial isolation from each pus sample was carried out on both MacConkey agar (Oxoid) and blood agar (Oxoid). A total of about 64 bacterial isolates were obtained. All of these isolates were assayed for antibiotic sensitivity using 13 antibiotics that assimilate 7 antibiotic types such as cephalosporines, polymexins, penicillins, fluroquinolones, aminoglycosides, macrolides, and sulphonamides [19,20,69]. Results were taken according to CLSI [70]. Multiple antibiotic resistance index (MAR) was calculated using the following formula: MAR index = number of antibiotics to which the bacterial isolate were resistant/Total number of antibiotics investigated [11].

The MDR bacteria obtained were the isolates DFU 9, 11, 16, and 32. They were identified biochemically using the Vitek-2 system [71]. The bacterial strain DFU 11, with the highest MAR index, was subjected to molecular identification for full characterization and to confirm its identification by fingerprinting of the sequence of 16S rRNA gene. Therefore, its DNA was extracted. Then, its 16S rRNA gene was amplified by polymerase chain reaction using universal primers: forward primer; 5-AGAGTTTGATCCTGGCTCAG-3′ and reverse primer, 3′GGTTACCTTGTTACGATT-5′. The PCR was carried out in a Gen Amp PCR system 9600 thermocycler (Perkin Elmer Company, NJ, USA) as described previously [72]. The PCR product was electrophoresed using agarose gel (0.7%) (Gomhuria Company., Egypt). The 16S rRNA gene band that appeared at 1500 bp was cut by Gene purification kit (Promega, Corporation, Madison, WI, USA) and the nucleotide sequence of 16S rRNA gene of the strain DFU 11 was sequenced by using a 3130x DNA sequencer (Genetic Analyser, Hitachi, Ibasaki, Japan). The sequence was submitted to the Gene Bank at http://BLAST.NCBI.NLM.NEH.gov (accessed on 2 March 2022) and by using the Basic Local Alignment Search Tool Program. A cluster analysis and phylogenetic tree were constructed (Supplement Figure S1). It was shown that the bacterial isolate DFU 11 showed similarity to the *Proetus mirabilis* cluster by >99.6% and consequently, an accession number LC587231 was given to the strain DFU 11 and was identified as belonging to *Proteus mirabilis*, and designated *P. mirabilis* LC587231 (*P. mirabilis*).

### 4.3. Preparayion of the Clove Flower Extract CFE and Phytochemical Screening

CFE was prepared as described previously [73,74], Briefly, 500 gm of clove flower buds were cleaned from the impurities and washed by distilled water, then dried in a hot air oven (Alexandria Co., Alexandria, Egypt) for 24 h at 40 °C. The flowers were then grounded to powdered form using a clean sterile mortar and pestle (Moulinex, Cairo, Egypt) and packed in an airtight plastic container until used. About 10 g aliquots of the powdered clove flower buds were extracted with 1000 mL ethanol by maceration, then filtered through a Buchner funnel with Whatman filter paper number 3, then evaporated under reduced pressure to dryness at 45 °C. A stock preparation (1 g/50 mL) of CFE was suspended in 1% DMSO and sterilized by Milipore filtration (0.45 μm, Amicon). The plant phytochemical screening was done with GC-Mass [29]. A direct capillary column TG-5MS (30 m × 0.25 mm × 0.25 μm thickness) was used. About 3 μL of CFE was injected automatically to the equipment using an Auto sampler AS3000 coupled with GC in the split mode. Then the instrumental analysis was carried out as described previously [11]. The components were identified by comparison of their retention times and mass spectra with those of Wiley 09 and NIST 11 mass spectra databases [11].

### 4.4. Antibacterial Activity of CFE against MDR P. mirabilis

Muller-Hinton agar plates (Oxoid) were prepared and seeded with log phase cells of *P. mirabilis* (10^5^ CFU/mL). Then, 5 mm wells were created with a sterile crok pourer and loaded with 100 μL of CFE. Finally, the plates were incubated for 24 h at 37 °C and the inhibition zones were measured in millimeters. The antibacterial activity was done in triplicates to confirm the antibacterial activity [11].

### 4.5. (MICs) of Both CFE and the Antibiotic Cefepime

MIC of CFE was determined as described previously [28]. Briefly, serial two-fold dilutions were made from 1 mL CFE in sterile test tubes containing brain heart infusion broth (BHI, Oxoid). The tubes were inoculated with 2 × 10^5^ CFU/mL of *P. mirabilis*, and were then incubated at 37 °C for 48 h. The last dilution with no visible growth (no turbidity) was defined as the MIC. From the last two tubes giving growth inhibition, 100 μL was streaked onto BHI agar and incubated at 37 °C for 24 h. The last concentration that showed no growth was defined as the minimum concentration that killed bacterial cells (MBC).

MIC of the antibiotic cefepime was determined according to Abdel-Shafi et al. [39]. Serial two-fold dilutions of the antibiotic cefepime (1 g/50 mL, Pharco, Pharmacia) were made in sterilized distilled water (100 μL, Eppendorf) (65) and were added to brain heart infusion plates, and then the plates were inoculated with about 2 × 10^5^ CFU/mL of actively growing cells of *P. mirabilis* and incubated at 37 °C for 24 h. MIC and MBC were determined as described previously [11,28].

### 4.6. CFE and Cefepime Hydrogel Preparation

The hydrogel was used as a vehicle for topical application of either CFE or the antibiotic cefepime that was formed as described previously [75,76,77]. In a sterile packed jar containing 50 mL sterile distilled water, sodium methylparaben, sodium propylparaben and sodium metabisulfite (about 4 g of each) were dissolved. Then, carbopol was added gradually with stirring slowly thoroughly, with addition of either CFE or the antibiotic cefepime until a swollen gel was developed. The whole volume was completed by distilled water to create a 100 g gel that contained 20 mg/mL of either CFE or the antibiotic cefepime. The jar containing the prepared gel was covered with a screw-capped plastic lid and was left for 24 h at room temperature to allow components to equilibrate; then, they were kept in refrigerator until used.

### 4.7. Experimental Animals and Ethical Declaration

The Zagazig University Animal Care Board approved the experiments carried on animals (*Rattus norvegicus*). Forty-five male adult mature albino rats (*Rattus norvegicus*) 8–10 weeks old and weighing 250–300 gm were purchased from Lab. Animal House, Faculty of Veterinary Medicine Zagazig University, Zagazig, Egypt. Rats were housed separately in a polypropylene rat cage with standard housing conditions: relative humidity 45–50%, 12 h light/dark cycles, and temperature 22 ± 2 °C. Rats were fed on a standard pelleted diet *ad-libitum* with free access to water throughout the experimental period. Before any experimental procedures, lab animals were left one week for acclimatization. 

### 4.8. Experimental Design

Forty-five male adult albino rats (*Rattus norvegicus*) were intraperitoneally injected with freshly prepared STZ in 0.1 M citrate buffer at a dose of about 65 mg/kg body weight for Type 1 diabetes induction as described previously [78]. Seven days post STZ injection, the Type 1 diabetes onset was assessed via measuring fasting blood glucose using a digital glucometer (U-Right, Korea). Rats that had blood glucose levels above 350 mg/dL were enrolled in the experiment. Then, wounds were done in accordance with Muhammad et al. [79]. In brief, rats were anesthetized with an intraperitoneal injection of ketamine 90 mg/kg and xylazine 10 mg/kg. Dorsal fur was clipped with an electrical hair clipper (Alexandaria Company, Egypt), the skin was disinfected with 70% ethanol, a full-thickness round wound excision 10 mm in diameter and 2 mm in thickness was created with Biopsy Punch (Gomhuria Company, Egypt), and then infected with 2 × 10^8^ CFU/mL of the *P. mirabilis* bacterium. After two days of infection, the rats were divided into three groups each of 15 rats. In group 1, the diabetic infected wounds of this group were treated with cefepime hydrogel. In group 2, the diabetic infected wounds were treated with CFE hydrogel; in group 3, diabetic infected wounds were treated with a hydrogel only (control). The treatment of diabetic wounds with hydrogels mentioned above were carried out one time every day throughout two weeks. Rats were kept separately in polypropylene cages to avoid fighting and wound biting. To assess the wound healing capacity, wound diameter and wound index were evaluated every 3 days throughout 18 days. At the end of the experiment, blood was collected from retro-orbital eye saphenous. Then, rats were killed and the skin of the wound was excised and divided into four parts; one part was collected on 10% formalin neutral buffer for histopathological examination, the second part was collected in peptone water for determining wound microbial load, the third part was stored at −20 °C for oxidant/antioxidant activity, and the fourth part, being 24 h of diabetic skin wound, was collected in 1 mL of quiazol (Qiagen, Germany) for total RNA extraction for RT-qPCR to quantify relative diabetic wound gene expression.

### 4.9. Measuring Glycemic Parameters and Oxidant/Antioxidant Activity

Blood glucose was measured 7 days post-STZ injection with a digital glucometer (U-Right, Korea). Insulin was measured in serum with sandwich ELISA Kit (SunRedBio, China) as described previously [80]. Lipid peroxidation marker Malondialdhyde *MDA) and antioxidant enzyme glutathione peroxidase (GPx), common antioxidant enzyme (CAT), and superoxide dismutase (SOD) were locally measured with a sandwich ELISA kit (SunRedBio, China) according to the method developed by Zhang et al. [81].

### 4.10. Measuring Wound Diameter and Wound Index

Wound diameter was measured with a measuring scale per centimeter and the wound index was calculated with the following equation [wound diameter of each time set point/initial wound diameter] according to Mendes et al. [37].

### 4.11. Rt-qPCR for Wound Gene Expression

Total RNA was extracted from 50 mg skin tissue with 1 mL Quiazol (QIAGEN, Germany) according to the manufacturer’s instruction. The extracted total RNA was measured with a NanoDrop^®^ ND-1000 UV-Vis Spectrophotometer (Thermo Scientific, Waltham, MA, USA) at wavelengths of 260 and 280 nm. The ratio of OD 260/280 was calculated to determine the quality of the extracted RNA; ones that were accepted for gene expression were located in the value between 1.8–2. cDNA was synthesized from 1 µg with a high capacity reverse transcriptase kit (Applied Biosystem, Foster City, CA, USA) in a final reaction volume of 20 µL (10 µL master mix and 10 µL RNA sample containing 1 µg RNA). cDNA was diluted 1:10, stored in aliquots at −20 °C for qPCR reaction. The qPCR was done by a real-time thermal cycler, Rotor-Gene Q 2 plex (Qiagen, Germany), as described previously [82]. Briefly, starting with a final volume reaction of 20 µL, using TOPreal syberGreen (Enzynomics, Korea) 10 µL, 1 µL of each forward and reverse primers synthesized by Sangon Biotech (Beijing, China) (Table 4), 1 µL of 1:10 diluted cDNA, and up to 20 µL of nuclease-free water were added, with cycling conditions of initial denaturation 95 °C for 10 min, 40 cycles of denaturation at 95 °C for 10 s, annealing at 60 °C for 15 s, and extension 72 for 15 s, and a melt curve analysis was performed. The gene expression was measured as relative fold change to an internal control reference gene (*Gapdh)* as described previously [83]. Briefly, Δ ct was calculated as the ct difference between the target gene and reference gene, then, ΔΔ ct was calculated as the difference between Δ ct of the sample and the average Δ ct of the control; finally, fold gene of the gene expression was calculated as 2^−(ΔΔct)^.

### 4.12. Determination of the Wound Total Bacterial and Coliform Count

The total bacterial and coliform count were carried out according to Xu et al. [84]. Briefly, 1 gm of diabetic wound skin was macerated in 9 mL peptone water and serially diluted with ten-fold dilution. From each dilution, 100 µL was cultured on both nutrient and McConkey agar (Difco) with the spread plate method and incubated at 37 °C for 24 h. Then, the counting of the colony-forming unit (CFU) for either total bacterial or coliform count were performed in triplicates. 

### 4.13. Histopathological Examination for H&E and Masson Blue

The histopathological procedures were done in accordance with Bancroft and Layton [85]. In brief, at the end of the experiment, the sacrificed skin of the wound area of rats was rapidly removed and collected in 10% formalin neutral buffer then embedded in a paraffin wax block and cut into 4 µm slices, before being mounted on a microscope slides that were used for histological stains. Before any histopathological procedures, the slides were incubated in xylene and passaged in ethanol with different concentrations for rehydration and paraffin wax removal, then the slides were stained with H&E for assessment of wound healing and Masson Trichome for collagen deposition (percentage of collagen deposition to control group was analyzed with Fiji software (http://fiji.sc, accessed on 28 June 2012). 

### 4.14. Data Analysis and Statistics

Statistical analysis was performed by GraphPad Prism 8 software (GraphPad Software Inc., San Diego, CA, USA). Data are expressed as mean ± standard error mean (S.E.M). Statistical comparisons were performed using a one-way analysis of variance (ANOVA) test followed by a post hoc Tukey test. The results indicated a statistical significance when *p* < 0.05.

## 5. Conclusions

It was concluded from this work that in diabetic foot infections, bacteria causing DFU can resist the action of many antibiotics and could cause many complications. CFE was shown to be a promising antibiotic alternative when used in hydrogel topical applications, as it improved the healing of STZ-induced diabetic rat wounds infected with multidrug resistant bacteria; its effect almost matched the one caused by cefepime and was superior in improving wound size, wound index, and certain immunological markers. However, further work will be necessary to investigate the in vitro and in vivo synergistic effect of both CFE and the antibiotic cefepime in combinations.

## Figures and Tables

**Table 1:** Selection and Identification of antibiotic-resistant isolates.

No	Biochemical Tests	Abbreviations	*Morganella monganii*	*Proteus mirabilis*	*Serratia fonticola*	*Escherichia coli*
1-	Ala-phe-pro-arylamidase	APPA	−ve	−ve	−ve	−ve
2-	Hydrogen sulfide production	H2S	+ve	+ve	+ve	−ve
3-	Beta-glucosidase	BGLu	−ve	−ve	+ve	−ve
4-	L-proline arylamidase	ProA	−ve	−ve	−ve	+ve
5-	Saccharose/Sucralose	SAC	−ve	−ve	+ve	+ve
6-	L-Lactate alkalinization	LLATK	+ve	+ve	+ve	+ve
7-	Glycine arylamidase	GlyA	−ve	−ve	−ve	−ve
8-	O/129 Resistance	O129R	+ve	+ve	+ve	+ve
9-	Adonitol	ADO	−ve	−ve	+ve	−ve
10-	Beta-*N*-Acetyl-glucose aminidase	BNAG	−ve	−ve	−ve	−ve
11-	D-Maltose	dMAL	−ve	−ve	+ve	+ve
12-	Lipase	LiP	−ve	−ve	−ve	−ve
13-	d-Tagatose	dTAG	−ve	−ve	−ve	−ve
14-	Alpha-glucosidase	AGlu	−ve	−ve	+ve	−ve
15-	Ornithine decarboxylase	ODC	+ve	+ve	−ve	−ve
16-	Glu-Gly-Arg-Arylamidase	GGAA	−ve	−ve	−ve	−ve
17-	L-pyrrolydonyl-arilamidase	PyrA	−ve	−ve	−ve	−ve
18-	Glutamyl arylamidase PNA	AGLTP	−ve	−ve	−ve	−ve
19-	D-Mannitol	dMAN	−ve	−ve	+ve	+ve
20-	Palatinose	PLE	−ve	−ve	+ve	−ve
21-	D-Trehalose	dTRE	−ve	+ve	−ve	+ve
22-	Succinate alkalinization	SuCT	+ve	+ve	+ve	+ve
23-	Lysine decarboxylase	LDC	−ve	−ve	−ve	+ve
24-	I-Malate assimilation	IMLTa	−ve	−ve	−ve	−ve
25-	L-Arabitol	lARL	−ve	−ve	+ve	−ve
26-	D-Glucose	dGLu	+ve	+ve	+ve	+ve
27-	D-Mannose	dMNE	+ve	−ve	+ve	+ve
28-	Tyrosine arylamidase	TyrA	+ve	+ve	+ve	+ve
29-	Citrate (Sodium)	CIT	−ve	+ve	+ve	−ve
30-	Beta-N-Acetyl-galactosaminidase	NAGA	−ve	−ve	−ve	−ve
31-	L-histidine assimilation	LHISa	−ve	−ve	−ve	−ve
32-	ELLMAN	ELLM	+ve	+ve	+ve	−ve
33-	D-Cellobiose	dCEL	−ve	−ve	−ve	−ve
34-	Gamma-Glutamyl transferase	GGT	+ve	+ve	+ve	−ve
35-	Beta-Xylosidase	Bxyl	−ve	−ve	−ve	−ve
36-	Urease	URE	+ve	+ve	+ve	−ve
37-	Malonate	MNT	−ve	−ve	−ve	−ve
38-	Alpha-galactosidase	AGAL	−ve	−ve	−ve	+ve
39-	Coumarate	CMT	+ve	+ve	+ve	+ve
40-	L-lactate assimilation	ILATa	−ve	−ve	−ve	−ve
41-	Beta-galactosidase	BGAL	−ve	−ve	−ve	+ve
42-	Fermentation glycose	OFF	+ve	+ve	+ve	+ve
43-	Beta-alanine arylamidase PNA	BALaP	−ve	−ve	−ve	−ve
44-	D-Sorbitol	dSOR	−ve	−ve	−ve	+ve
45-	5-Keto-D-gluconate	5KG	−ve	−ve	−ve	−ve
46-	Phosphatase	PHOS	+ve	+ve	+ve	−ve
47-	Beta-Glucoronidase	BGUR	−ve	−ve	−ve	−ve

**Table 2:** Minimum inhibitory concentrations (MICs) of the antibiotic cefepime and clove flower extract (CFE).

Concentration of the Antimicrobial Agents	Growth of *P. mirabilis*
Cefepime (μg/mL)	
10	Positive
20	Positive
30	Positive
40	Positive
50	No growth
CFE (μg/mL)	
2	Positive
4	Positive
6	Positive
8	Negative
10	Negative

**Table 3:** GC-Mass analysis report for the clove ethanolic extract.

RT/min	Name & Class	Mol. Formula	Mol. wt	Area	Base Peak (100%)
**13.78**	Eugenol (Phenol)	C_10_H_12_O_2_	164.0	82.34	77.00
**14.40**	Caryophyllene (Bicyclic)	C_15_H_24_	204.0	3.20	133.0 & 93.00
**16.47**	3-Allyl-6-methoxyphenyl acetate	C_15_H_24_	204.0	0.26	161.0
**17.04**	Phenol, 2-methoxy-4-(2-propenyl)-, acetate	C_12_H_14_O_3_	206.0	0.24	164.0
**17.79**	Caryophyllene oxide (Bicyclic)	C_15_H_24_O	220.0	0.48	41.00
**19.06**	Caryophylla-4(12),8(13)-dien-5à-ol	C_15_H_24_O	220.0	0.42	136.0
**20.15**	Benzene, 1,4-dimethyl-2-[(3-methylphenyl)methyl]	C_16_H_18_	210	0.12	195
**43.05**	Butanoic acid, 3-methyl-, 2-methoxy-4-(2-propenyl)phenyl ester	C_15_H_20_O_3_	248.0	0.27	164.0
**49.51**	α-sitosterol (steroid)	C_29_H_50_O	414.0	0.84	107.0

**Table 4:** Primers sequence used and their sequences.

Primer	Forward Primer (5′–3′)	Reverse Primer (5′–3′)	Size	Accession No.
***PCNA***	ATCTAGACGTCGCAACTCCG	GCTGCACTAAGGAGACGTGA	173	NM_022381.3
***Mmp9***	GATCCCCAGAGCGTTACTCG	GTTGTGGAAACTCACACGCC	132	NM_031055.2
***Collagen***	GCAATGCTGAATCGTCCCAC	CAGCACAGGCCCTCAAAAAC	176	NM_053304.1
***Fibronectin***	GGATCCCCTCCCAGAGAAGT	GGGTGTGGAAGGGTAACCAG	188	NM_019143.2
***NF-κβ1***	CCACTGTCAACAGATGGCCC	CTTTGCAGGCCCCACATAGT	177	NM_001276711.1
***TNF-α***	GGCTTTCGGAACTCACTGGA	GGGAACAGTCTGGGAAGCTC	164	NM_012675.3
***IL-10***	GCTCAGCACTGCTATGTTGC	TTGTCACCCCGGATGGAATG	76	NM_012854.2
***IL-8***	ACAGGCAGGCTGTAGTTGTC	ATCACCAGCGAGTTTCCCAG	70	NM_019310.1
***IL-4***	CGTGATGTACCTCCGTGCTT	GTGAGTTCAGACCGCTGACA	88	NM_201270.1
***IL-1β***	GAGTCTGCACAGTTCCCCAA	TCCTGGGGAAGGCATTAGGA	158	NM_031512.2
***MCP-1***	TAGCATCCACGTGCTGTCTC	CAGCCGACTCATTGGGATCA	94	NM_031530.1
***TGF-β1***	AGGGCTACCATGCCAACTTC	CCACGTAGTAGACGATGGGC	168	NM_021578.2
***IL-1β***	GAGTCTGCACAGTTCCCCAA	TCCTGGGGAAGGCATTAGGA	158	NM_031512.2
***Gapdh***	GCATCTTCTTGTGCAGTGCC	GGTAACCAGGCGTCCGATAC	91	NM_017008.4
***GLP1***	CACCTCCTCTCAGCTCAGTC	CGTTCTCCTCCGTGTCTTGA	128	NM_012707.2
***GLPr1***	CTTGGAGACATAGAAGGGGGAC	AGGAGCATGCCTCTGGGTAG	128	NM_172091.2
***EGF-β1r***	GGCATCATGGGGGAGAACAA	GGATCTTTGGCCCATAGGTACAG	100	NM_001393707.1
***EGF-β1***	GGTCCACCCATTGGCAAAAC	CACGAATCCTTCCCGACACA	118	NM_012842.2
***PPAR-α***	GTCCTCTGGTTGTCCCCTTG	GTCAGTTCACAGGGAAGGCA	176	NM_013196.2
***PGC-1α***	TTCAGGAGCTGGATGGCTTG	GGGCAGCACACTCTATGTCA	70	NM_031347.1
***FGF***	GAGCGACCCTCACATCAA	CGTTTCAGTGCCACATACC	222	NM_019305.2
***VEGF***	GATCCAGTACCCGAGCAGTCA	TCTCCTTTCTTTTTGGTCTGCAT	83	NM_053549.1
***Bax***	CGAATTGGCGATGAACTGGA	CAAACATGTCAGCTGCCACAC	109	NM_017059.2
***Bcl-2***	GACTGAGTACCTGAACCGGCATC	CTGAGCAGCGTCTTCAGAGACA	135	NM_016993.1
***Fas***	GAGCGTTCGTGAAACCGACA	AGGTTGGTGCACCTCCACTTG	128	NM_139194.2
***FasL***	CACCAACCACAGCCTTAGAGTATCA	CACTCCAGAGATCAAAGCAGTTCC	172	NM_012908.1
***P38***	CGGCTTGCTCATGTCCTCAGAAC	GGAGGGCGGCTGCACATACAC	214	NM_031020.2
***P53***	CATGAGCGTTGCTCTGATGGT	GATTTCCTTCCACCCGGATAA	67	NM_030989.3
***Casp-3***	GAGACAGACAGTGGAACTGACGATG	GGCGCAAAGTGACTGGATGA	147	NM_012922.2
***16s rRNA***	AGAGTTTGATCCTGGCTCAG	CTACGGCTACCTTGTTACGA	1507	NR_043997.1

## Data Availability

The data that support the findings of this study are available from the corresponding author upon reasonable request.

## Supplementary Materials

The following supporting information can be downloaded at: https://www.mdpi.com/article/10.3390/molecules27072270/s1. Table S1: Susceptibility of bacterial isolates to different antibiotics; Figure S1: Phylogenetic tree analysis based on 16 s rRNA nucleotide sequence alignment for *P. mirabilis* with other related members that possess the best similarity; Figure S2: GC-Mass of the *Syzygium aromaticum* ethanolic extract indicating retention time of different identified bioactive compounds; Figure S3: GC-Mass fragmentation pattern of the different identified bioactive compounds from the ethanolic extract of the *Syzygium aromaticum* indicating molecular weight (*m*/*z*) of each compound.

## Sample Availability

Samples of the compounds aren’t available from the authors.
