# Supplementary material for: The Healing Capability of Clove Flower Extract (CFE) in Streptozotocin-Induced (STZ-Induced) Diabetic Rat Wounds Infected with Multidrug Resistant Bacteria
Source: Molecules. 2022 Mar 31;27(7):2270. doi: 10.3390/molecules27072270 (PMC9000752; doi:10.3390/molecules27072270)
Supplement: Supplementary file 1 [file molecules-27-02270-s001.zip › molecules-1644578-supplementary.pdf]

Supplementary Table S1. Susceptibility of bacterial isolates to different antibiotics.

Isolate	Cephalosporins				Polymyxins	Penicillins			Fluoroquinolone	Aminoglycoside		Macrolides	Sulfonamide	MAR
No.	FOX_{30}	FEP_{30}	CAZ_{30}	CRO_{30}	PB_{300}	PRL_{100}	IPM_{10}	AMC_{30}	CIP_{5}	AK_{30}	CN_{10}	E_{15}	SXT_{25}	index (%)
DFU1	20.00	22.00	14.00	19.00	12.00	18.00	27.00	20.00	19.00	20.00	17.00	22.00	26.00	0.00
DFU2	20.00	10.00	12.00	10.00	0.00	15.00	20.00	12.00	20.00	13.00	8.00	20.00	20.00	7.70
DFU3	25.00	8.00	10.00	22.00	14.00	10.00	28.00	28.00	10.00	16.00	17.00	15.00	20.00	0.00
DFU4	23.00	20.00	17.00	15.00	13.00	25.00	21.00	10.00	28.00	18.00	15.00	10.00	25.00	0.00
DFU5	24.00	15.00	15.00	20.00	0.00	12.00	17.00	10.00	30.00	13.00	15.00	0.00	20.00	15.4
DFU6	18.00	25.00	17.00	30.00	0.00	20.00	15.00	23.00	30.00	15.00	20.00	0.00	18.0	15.4
DFU7	12.00	0.00	15.0	20.00	0.00	10.00	10.00	12.00	25.00	18.00	8.00	0.00	0.00	30.7
DFU8	15.00	12.00	15.00	18.00	15.00	15.00	18.00	14.0	26.00	15.00	10.00	0.00	0.00	15.40
DFU9	22.00	0.00	0.00	0.00	0.00	10.00	13.00	12.00	0.00	15.00	8.00	0.00	0.00	53.8
DFU10	12.00	12.00	20.00	30.00	18.00	15.00	25.00	28.00	0.00	20.00	10.00	0.00	0.0	23.1
DFU11	0.00	0.00	20.00	12.00	12.00	0.00	25.00	18.00	0.00	20.00	11.00	0.00	0.00	46.2
DFU12	19.00	7.00	0.00	8.00	0.00	0.00	18.00	29.00	8.00	13.00	0.00	0.00	0.00	46.2
DFU13	10.00	0.00	12.00	12.00	0.00	12.00	16.00	0.00	17.00	12.00	0.00	0.00	0.00	46.1
DFU14	22.00	30.00	21.00	0.00	0.00	0.00	17.00	30.00	20.00	15.00	7.00	8.00	7.00	23.1
DFU15	25.00	29.00	30.00	28.00	11.00	35.00	25.00	42.00	0.00	27.00	12.00	17.00	18.00	7.7
DFU16	18.00	35.00	38.00	31.00	17.00	17.00	21.00	15.00	18.00	18.00	7.00	0.00	0.00	15.4
DFU17	14.00	35.00	40.00	25.00	0.00	15.00	12.00	14.00	20.00	10.00	10.00	0.00	0.00	23.1
DFU18	10.00	12.00	14.00	10.00	14.00	15.00	17.00	20.00	12.00	17.00	0.00	15.00	0.00	15.4
DFU19	20.00	10.00	10.00	15.00	18.00	25.00	26.00	26.00	20.00	20.00	16.00	12.00	25.00	0.00
DFU20	15.00	0.00	0.00	12.00	15.00	20.00	11.00	16.00	0.00	13.00	12.00	15.00	0.00	30.8
DFU21	15.00	16.00	15.00	12.00	10.00	0.00	17.00	11.00	18.00	16.00	0.00	0.00	0.00	30.8
DFU22	15.00	15.00	10.00	15.00	10.00	12.00	15.00	20.00	17.00	15.00	15.00	0.00	25.00	7.7
DFU23	15.00	15.00	13.00	15.00	0.00	14.00	12.00	10.00	17.00	12.00	0.00	0.00	11.00	23.1
DFU24	14.00	0.00	15.00	14.00	12.00	10.00	10.00	0.00	0.00	15.00	20.00	0.00	0.00	38.5
DFU25	26.00	13.00	15.00	11.00	15.00	12.00	18.00	17.00	26.00	12.00	12.00	0.00	22.00	7.7
DFU26	20.00	0.00	10.00	0.00	10.00	15.00	20.00	15.00	22.00	20.00	17.00	0.00	22.00	23.1
DFU27	25.00	0.00	15.00	0.00	0.00	17.00	20.00	15.00	23.00	0.00	0.00	25.00	22.00	38.4
DFU28	26.00	11.00	12.00	0.00	0.00	17.00	22.00	15.00	22.00	0.00	0.00	22.00	22.00	30.8
DFU29	20.00	0.00	0.00	0.00	20.00	0.00	25.00	12.00	0.00	15.00	14.00	0.00	0.00	53.8*
DFU30	15.00	18.00	14.00	15.00	0.00	0.00	18.00	15.00	28.00	18.00	11.00	0.00	0.00	30.8
DFU31	12.0	15.00	13.00	20.00	10.00	13.00	16.00	12.00	30.00	17.00	10.00	0.00	0.00	15.4
%R	0.0	7.00	4.00	6.00	13.00	6.00	0.00	2.00	7.00	20.00	7.00	20.00	16.00	
	00%	29%	16%	25%	54%	25%	0.00%	8.3%	29%	8.3%	29%	83.3%	66.7%	

AK_{30}: Amikacin, AMC_{30}: Amoxicillin, CAZ_{30}: Ceftazidime, CIP_{5}: Ciprofloxacin, CN_{10}: Gentamicin, CRO_{30}: Ceftriaxone, E_{15}: Erythromycin, FEP_{30}: Cefepime, FOX_{30}: Cefoxitin, IPM_{10}: Imipenem, PB_{300}: Polymyxin, PRL_{100}: Piperacillin, SXT_{25}: Trimethoprim-sulphamethoxaol.

Supplementary Table S(1): Continued.

Isolate No.	Cephalosporins				Polymyxins	Penicillins			Fluoroquinolone	Aminoglycoside		Macrolides	Sulfonamide	% Resist-ance
	FOX_{30}	FEP_{30}	CAZ_{30}	CRO_{30}	PB_{300}	PRL_{100}	IPM_{10}	AMC_{30}	CIP_{5}	AK_{30}	CN_{10}	E_{15}	SXT_{25}	
DFU32	17.00	12.00	10.00	13.00	10.00	20.00	20.00	13.00	25.00	18.00	15.00	0.00	26.00	7.7
DFU33	0.00	0.00	0.00	0.00	12.00	0.00	10.00	0.00	0.00	15.00	0.00	0.00	0.00	76.9
DFU34	15.00	18.00	10.00	0.00	8.00	12.00	0.00	0.00	15.00	10.00	15.00	0.00	0.00	38.5
DFU35	15.00	0.00	15.00	0.00	18.00	10.00	7.00	0.00	25.00	15.00	0.00	12.00	0.00	38.5
DFU36	0.00	0.00	0.00	0.00	0.00	0.00	0.00	15.00	12.00	20.00	15.00	0.00	12.00	61.5
DFU37	11.00	0.00	0.00	0.00	0.00	0.00	11.00	8.00	16.00	0.00	0.00	0.00	0.00	69.2
DFU38	20.00	0.00	7.00	11.00	11.00	21.00	22.00	12.00	0.00	13.00	9.00	0.00	0.00	30.8
DFU39	17.00	0.00	0.00	12.00	0.00	13.00	8.00	14.00	4.00	15.00	0.00	0.00	0.00	46.2
DFU40	0.0	18.00	17.00	40.00	12.00	20.00	16.00	40.00	0.00	16.00	12.00	0.00	0.00	30.8
DFU41	18.00	12.00	10.00	18.00	11.00	16.00	15.00	15.00	20.00	18.00	15.00	0.00	0.00	15.4
DFU42	22.00	23.00	18.00	12.00	0.00	15.00	23.00	17.00	36.00	21.00	13.00	0.00	0.00	23.1
DFU43	18.00	12.00	0.00	9.00	0.00	20.00	12.00	17.00	0.00	12.00	7.00	0.00	8.00	30.8
DFU44	21.00	15.00	15.00	6.00	0.00	24.00	20.00	28.00	7.00	17.00	9.00	7.00	4.00	7.7
DFU45	28.00	20.00	19.00	36.00	40.00	0.00	18.00	38.00	28.00	18.00	9.00	6.00	8.00	7.7
DFU46	19.00	18.00	30.00	34.00	0.00	30.00	35.00	50.00	19.00	16.00	0.00	0.00	0.00	30.8
DFU47	15.00	0.00	0.00	8.00	0.00	15.00	11.00	12.00	15.00	0.00	16.00	0.00	10.00	38.5
DFU48	0.00	0.00	17.00	19.00	0.00	0.00	21.00	10.00	15.00	17.00	9.00	0.00	5.00	38.5
DFU49	10.00	10.00	15.00	8.00	0.00	9.00	18.00	17.00	25.00	15.00	7.00	0.00	0.00	23.1
DFU50	20.00	15.00	15.00	14.00	0.00	15.00	10.00	12.00	11.00	16.00	0.00	0.00	0.00	30.8
DFU51	15.00	28.00	17.00	15.00	20.00	13.00	20.00	10.00	12.00	15.00	7.00	0.00	0.00	15.4
DFU52	18.00	19.00	14.00	15.00	0.00	10.00	12.00	13.00	12.00	15.00	0.00	0.00	0.00	30.8
DFU53	17.00	0.00	0.00	0.00	12.00	0.00	23.00	11.00	0.00	14.00	13.00	12.00	16.00	38.5
DFU54	17.00	0.00	0.00	10.00	11.00	0.00	22.00	11.00	0.00	18.00	17.00	14.0	12.00	30.8
DFU55	17.00	0.00	15.00	0.00	0.00	8.00	17.00	10.00	0.00	15.00	0.00	0.000	22.00	46.2
DFU56	19.00	0.00	0.00	0.00	0.00	11.00	15.00	8.00	0.00	15.00	0.00	0.00	0.00	61.5
DFU57	15.00	15.00	17.00	18.00	0.00	15.00	16.0	12.00	17.00	12.00	0.00	0.00	0.00	30.8
DFU58	25.00	22.00	17.00	20.00	14.00	15.00	32.00	25.00	25.00	22.00	20.0	0.0	0.00	15.4
DFU59	20.00	22.00	15.00	20.00	10.00	9.00	31.00	16.00	22.00	16.00	18.00	0.00	16.00	7.7
DFU60	13.00	0.00	7.00	15.00	44.00	0.00	13.00	10.00	30.00	12.00	11.00	0.00	7.00	23.1
DFU61	9.00	8.00	0.00	0.00	19.00	0.00	0.00	10.00	0.00	0.00	17.00	0.00	0.00	61.5
DFU62	0.00	7.00	12.00	0.00	18.00	0.00	29.00	10.00	0.00	16.00	0.00	0.00	0.00	53.9
DFU63	15.00	25.00	18.00	31.00	0.00	0.00	17.00	0.00	18.00	15.00	19.00	0.00	0.00	38.5
DFU64	18.00	7.00	0.00	0.00	11.00	0.00	0.00	0.00	0.00	15.00	0.00	0.00	0.00	69.2
R%	12.2	36.6	34.14	34.14	46.30	34.14	9.80	12.2	31.7	7.31	39.0	75.60	61.01	

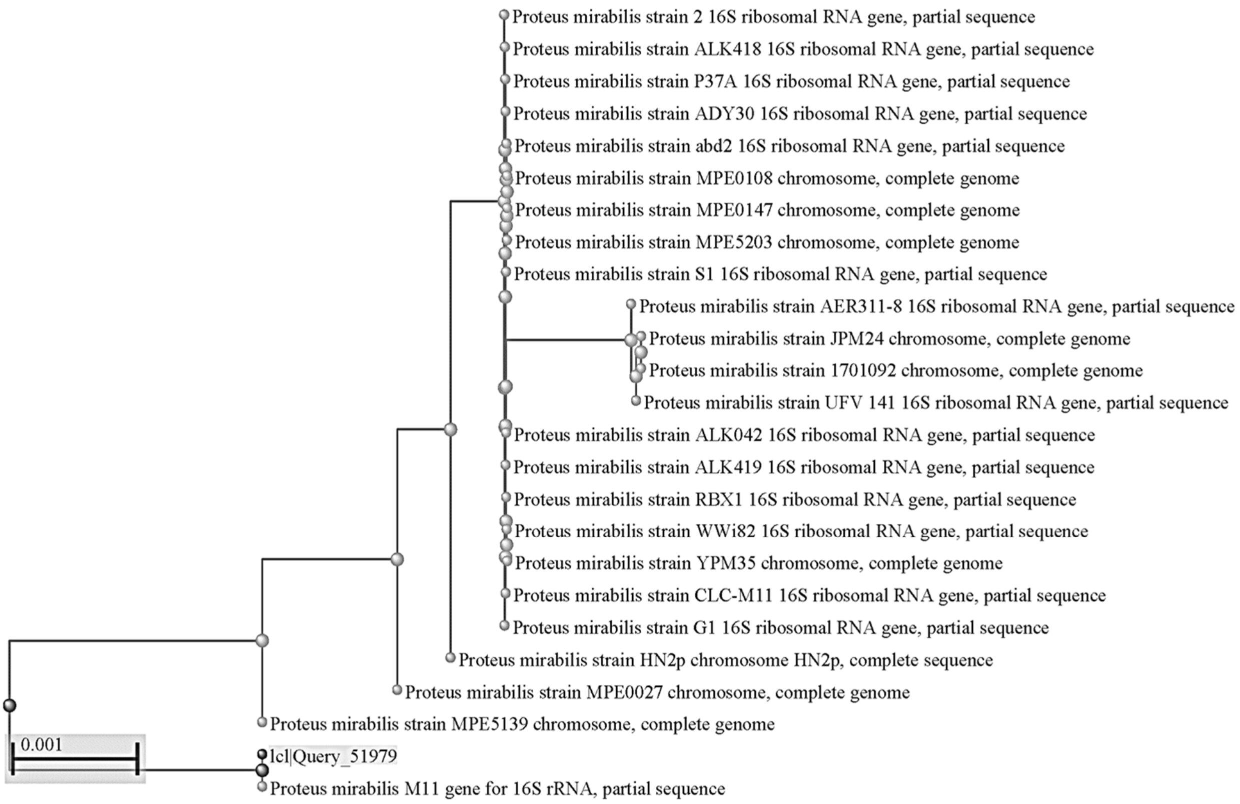

Supplementary Figure S1. Phylogenetic tree analysis based on 16 s rRNA nucleotide sequence alignment for *P. mirabilis* with other related members that possess the best similarity.

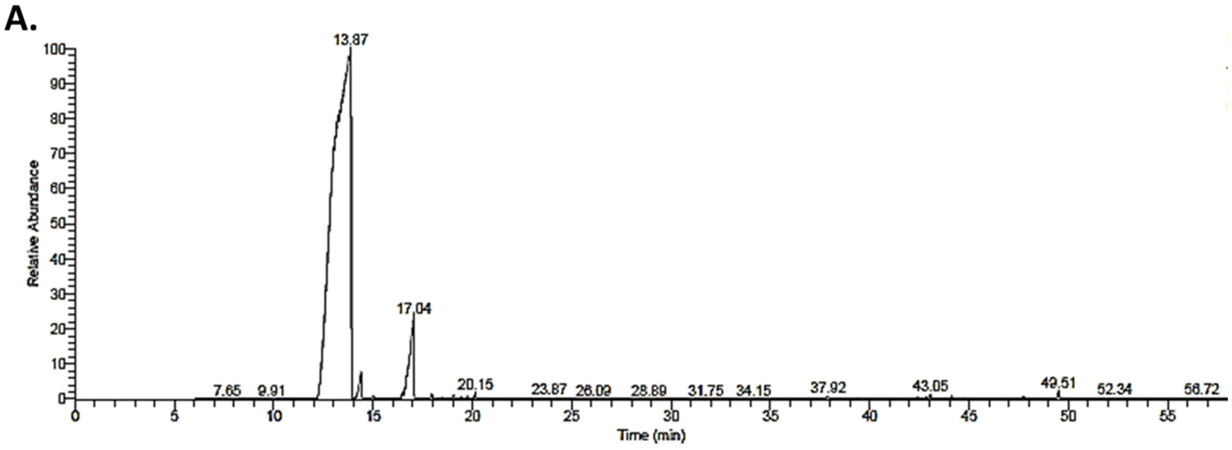

Supplementary Figure S2. GC-Mass of the *Syzygium aromaticum* ethanolic extract indicating retention time of different identified bioactive compounds.

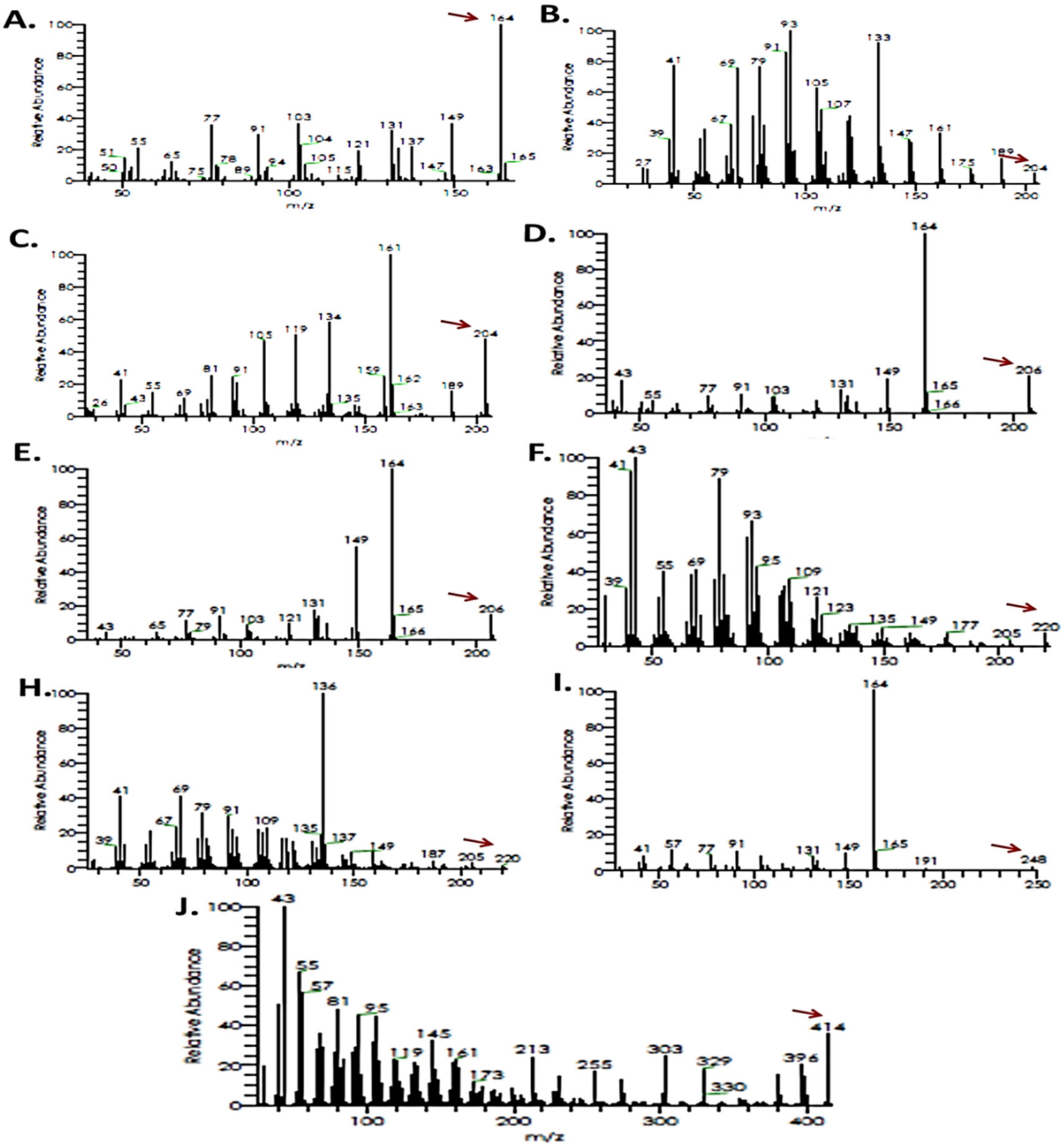

Supplementary Figure S3. GC-Mass fragmentation pattern of the different identified bioactive compounds from the ethanolic extract of the *Syzygium aromaticum* indicating molecular weight (m/z) of each compound (A. - I.) A. Eugenol (164 m/z), B. Caryophyllene (204 m/z), C. 3-Allyl-6-methoxyphenyl acetate (204 m/z), D. Phenol, 2-methoxy-4-(2-propenyl)-, acetate (206 m/z), E. Caryophyllene oxide (220 m/z), F. Caryophylla-4(12),8(13)-dien-5à-ol (220 m/z), G. Benzene, 1,4-dimethyl-2-[(3-methyl phenyl)methyl] (210 m/z), H. Butanoic acid, 3-methyl-, 2-methoxy-4-(2-propenyl)phenyl ester (248 m/z), I. ç-Sitosterol (414 m/z).
